# Nanotechnology-based radiation therapy to cure cancer and the challenges in its clinical applications

**DOI:** 10.1016/j.heliyon.2023.e17252

**Published:** 2023-06-13

**Authors:** Muhammad Arif, Ayesha Fazal Nawaz, Shahid Ullah khan, Hasnat Mueen, Fizza Rashid, Hassan A. Hemeg, Abdur Rauf

**Affiliations:** a Department of Plant Biology and Ecology, College of Life Sciences, Nankai University, Tianjin, PR China; b National Institute for Genomics and Advanced Biotechnology (NIGAB), National Agricultural Research Centre (NARC), Islamabad, Pakistan; c Department of Biochemistry, Women Medical and Dental College, Khyber Medical University KPK, Pakistan; d Department of Biotechnology, COMSATS University Islamabad, Abbottabad Campus, Abbottabad, Pakistan; e Department of Medical Laboratory Technology, College of Applied Medical Sciences, Taibah University, Al-Medinah Al-Monawara Postcode, Saudi Arabia; f Department of Chemistry, University of Swabi, Anbar 23561, Khyber Pakhtunkhwa, Pakistan

**Keywords:** Radiotherapy, Cancer, Nanotechnology, Radio-sensitizers, Nanoparticles

## Abstract

Radiation therapy against cancer frequently fails to attain the desired outcomes because of several restricting aspects. Radiation therapy is not a targeted antitumor treatment, and it poses serious threats to normal tissues as well. In many cases, some inherent features of tumors make them resistant to radiation therapy. Several nanoparticles have shown the capacity to upgrade the viability of radiation treatment because they can directly interact with ionizing radiation to increase cellular radiation sensitivity. Several types of nanomaterials have been investigated as radio-sensitizers, to improve the efficacy of radiotherapy and overcome radio-resistance including, metal-based nanoparticles, quantum dots, silica-based nanoparticles, polymeric nanoparticles, etc. Despite all this research and development, certain challenges associated with the exploitation of nanoparticles to enhance and improve radiation therapy for cancer treatment are encountered. Potential applications of nanoparticles as radiosensitizers is hindered by the difficulties associated with ensuring their production at a large scale with improved characterizations and because of certain biological challenges. By overcoming the shortcomings of nanoparticles like working on the pharmacokinetics, and physical and chemical characterization, the therapy can be improved. It is expected that in the future more knowledge will be available regarding nanoparticles and their clinical efficacy, leading to the successful development of nanotechnology-based radiation therapies for a variety of cancers. This review highlights the limitations of conventional radiotherapy in cancer treatment and explores the potential of nanotechnology, specifically the use of nanomaterials, to overcome these challenges. It discusses the concept of using nanomaterials to enhance the effectiveness of radiation therapy and provides an overview of different types of nanomaterials and their beneficial properties. The review emphasizes the need to address the obstacles and limitations associated with the application of nanotechnology in cancer radiation therapy for successful clinical translation.

## 1 Introduction

Every year, about 18 million new cases of cancer are reported with about 9.6 million fatalities [1]. According to the International Agency for Research on Cancer, by 2030 there will be 22.2 million new cases annually and the death toll is expected to be 13.2 million per year [2]. Current approaches to diagnostics and treatment are not enough to deal with this disease due to certain prominent limitations such as lack of specificity and selectivity which cause negative effects on normal cells [3]. Hence, new approaches need to be developed and investigated to complement current methods of diagnosis and treatment. It is believed that nanomedicine, which is based on nanotechnology, has the potential for the treatment of several fatal diseases including cancer.

Nowadays, nanotechnology is widely being investigated to achieve innovation in health care regarding the control, therapy, diagnosis, prevention, or monitoring of a medical condition, particularly cancer [4]. Extensive research has introduced a wide range of nanomaterials for cancer diagnosis and therapy [5]. Some of the cancer-related nanotechnologies have already been developed including injectable drug delivery nano-vectors [6]; methods based on nanoparticles for the detection of DNA and proteins with high-specificity; and biologically targeted, nanosized magnetic resonance imaging (MRI) agents for intraoperative imaging [7]. Apart from these, scientists are particularly interested in investigating the potential of nanotechnology in overcoming the challenges associated with conventional radiation therapies, used for the treatment of cancer.

The purpose of writing this review is to highlight the shortcomings of traditional radiotherapy in cancer treatment and discuss how nanotechnology, specifically the use of nanomaterials, can overcome these limitations and improve the effectiveness of radiation therapy. The review discusses the challenges faced in radiotherapy and the concept of using nanomaterials to enhance radiotherapy. By providing a detailed overview of different types of nanomaterials and their beneficial properties, this review aims to inform readers about the potential of nanotechnology in the field of radiation therapy. Finally, the review discusses the challenges associated with the application of nanotechnology in enhancing cancer radiation therapy and highlights various obstacles and limitations that need to be addressed for the successful clinical translation of nanotechnology-based radiation therapy.

## 2 Nanotechnology

Nanotechnology involves research regarding nanoparticles and technology development at the nanoscale i.e., at the atomic, molecular, or macromolecular level. The field aims to create devices, structures, and systems that have useful properties and functions. Alongside this, it also provides a better understanding of materials and phenomena at the nanoscale [8,9]. The use of nanotechnology has provided solutions to many medical problems, including smart nanomaterials for targeted drug delivery [10] bringing disruptive innovation to the field of oncology [11].

## 3 Radiation therapy

Radiation therapy (RT) involves the utilization of high doses of electromagnetic radiation to destroy tumors or cancerous cells in the body [12]. After the discovery and clinical exploitation of X-rays in cancer treatment, radiation therapy developed into a recognized medical specialty [13]. Radiation therapy is becoming particularly important in cancer treatment, along with chemotherapeutic and surgery approaches, as it is very cost-effective accounting for only 5% of the total cancer care cost [14]. About 50% of all patients suffering from cancer undergo radiation therapy during their course of treatment [15] and it is estimated that radiation therapy can contribute to around 40% of curative treatment [16].

The radiations used during therapy can kill the cancerous cells directly or destroy the ability of cancerous cells to proliferate further by damaging their genetic material [17]. Radiation therapy has been established as an important clinical modality for the treatment of several malignancies, such as lung carcinoma, gynecological cancers, thyroid carcinomas, central nervous system neoplasms, hematologic malignancies, breast carcinoma, melanoma, prostate tumors, gastrointestinal cancers, and cervix tumors [18]. Even though radiation therapy is widely used around the world for the treatment of cancer, it does possess many side effects as well [19].

**Fig. 1:**
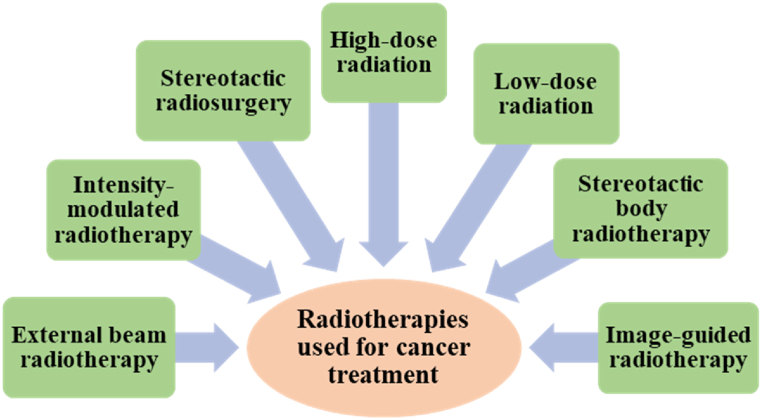
Currently being used radiation therapies for cancer treatment.

A number of radiation treatments are currently being used or developed for the treatment of cancer including External beam radiotherapy (EBRT), Intensity-modulated radiotherapy (IMRT), Image-guided radiotherapy (IGRT), Cone beam computer tomography (CBCT), Stereotactic body radiotherapy (SBRT), Stereotactic radiosurgery (SRS), High-dose radiation (HDR) and Low-dose radiation (LDR), etc. as documented in Fig. 1. EBRT involves all the clinical modalities that are based on radiation therapy using linear accelerators to provide safe and efficacious treatment against cancer [20]. IMRT is a radiotherapy treatment that, in order to decrease the exposure of radiation to other tissues, involves conforming radiation distribution shape to mimic the shape as well as the size of the tumor [21]. IGRT is a radiation therapy with improved and highly targeted delivery of radiation. In the past, tumors were irradiated using two-dimensional (2D) imaging without considering the protection of the normal tissues. As a result, even slight deviations or positional errors used to cause unwanted damage to healthy tissues [22]. IGRT, in contrast, can detect such errors by acquiring information through pre-radiotherapy imaging which allows for correction before the treatment begins [20]. SBRT is another relatively new approach that can be used to get rid of small, well-defined cancer tumors. It offers hypo-fractionated dose regimens which are given in less than five fractions which in turn improves local control and survival [23].

## 4 Nanotechnology-based radiotherapy

Nanotechnology has been widely used in cancer therapy and diagnosis. Although much of the research in nanotechnology for oncology is focused on diagnostics and chemotherapy delivery, research is also being carried out on applying nanotechnology to improve radiation oncology [24]. Although widely used, clinical radiotherapy still often fails to attain the desired outcomes regarding cancer treatment because of many constraining factors [25]. Nanotechnology, as an emerging multi-disciplinary field of science and technology, has become a promising research direction in cancer treatment. Nanomaterials, because of their extraordinary physical as well as chemical properties, have been used to enhance the effectiveness of radiation therapy and to overcome the tolerance of cancerous cells towards radiation [26].

### 4.1 Shortcomings of radiotherapy which are being overcome by nanotechnology

Radiation therapy, unlike several other therapeutic treatments, is not a selective therapeutic approach against cancer. A great challenge for the experts of radiation oncology, medical physics, and radiobiology is to enhance and improve the clinical efficacy of radiotherapy without damaging healthy tissues [27]. In the case of EBRT, high intensities of radiation are often required to effectively kill the tumors, as they absorb only a small proportion of the radiant energy tissue, posing a severe risk to health. Similarly, in internal radioisotope therapy (RIT) there are many chances of radio-toxicity to normal cells and tissues along with the cells of the tumor [28].

Despite the current advances in the fields of medical physics, radiobiology, and radiation oncology, radiation therapy responses vary greatly among individual patients. These variations in the response are usually because of the variations in the intrinsic resistance of tumor cells to radiotherapy that originates from either a different genetic background or altered protein expression. Moreover, the variations leading to variable responses are either already present in the genome of the patient and/or newly acquired malignant cells during cancer development [29]. Some cancer cells, farther from the site of application, receive a lower intensity of the radiation which is another disadvantage of radiotherapy [30].

The efficacy of radiotherapy is also greatly limited by some inherent characteristics of solid tumors. The microenvironment of cancerous cells is usually different from that of normal cells and has insufficient oxygenation, slightly acidic pH, asymmetrical distribution of both organic and inorganic nutrients, and a larger amount of reactive oxygen species (ROS) [31]. These characteristics of the tumor microenvironment greatly contribute to tumor invasion, metastasis, and recurrence. Moreover, these features also lead to resistance to several therapeutic strategies, including radiation therapy [25]. Due to tumor resistance to radiation, elevated doses of radiation are usually required which ultimately causes the death of the surrounding normal cells and tissues.

**Fig. 2:**
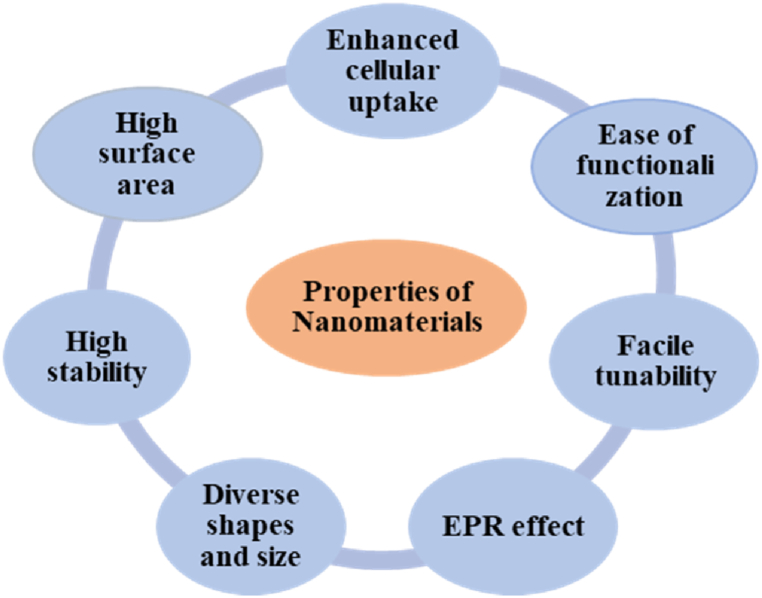
Unique properties of nanomaterials favoring scientists to devise novel and effective anticancer regimes.

Using nanoscale agents, radiation therapy can be made more effective. Scientists are taking great interest in achieving improved radiotherapy-based cancer treatment by using nanomaterials to enhance the response of cancerous cells towards radiation and overcome the radio resistance of tumors [32]. To devise novel and effective anticancer regimes, researchers are applying the unique characteristics of nanomaterials, which are documented in Fig. 2, including enhanced cellular uptake [33]; ease of functionalization [34]; unusual optical, electronic, and magnetic properties [35]; distinct biodistribution and pharmacokinetics; enhanced permeability and retention (EPR) effect; controlled release [36]; high surface area; stability; modifiable features including size, shape, and surface charge; and facile tunability to combat the challenges posed by classical radiation therapy [37].

A major and important aim of combining classical radiation therapy with nanotechnology is to increase the differential effect between cancerous and normal cells ensuring that only the tumors get affected by the application of the radiation [27]. Scientists are focusing on targeting cancer cells specifically while applying the radiation and sensitizing the radioresistant cancerous cells to conventional doses of radiation [38]. Several nanoparticles can play a role in increasing cellular radiation sensitivity by directly interacting with ionizing radiation. Such nanoparticles are usually based on elements with high atomic mass (A) and atomic number (Z) e.g., gold, silver, titanium, platinum, etc. [29]. Nanoparticles are not only used as radiosensitizers but can also be used as radioprotective agents to mitigate the harmful effects of radiation on healthy tissues. For example, nanoparticles containing antioxidants or free radical scavengers can protect normal cells from radiation-induced damage [39].

### 4.2 Nanomaterials used in cancer radiation therapy

Nanomaterials have shown the capacity to upgrade the viability of radiation treatment because of their capacity to concentrate the ionization energy inside cancer tumors [40]. The process of improving the vulnerability of tumors to injury by radiation is radiosensitization and radiosensitizers are agents that enhance the impacts of radiation on the cancerous cells [30]. A radiation sensitizer results in a differential impact on ordinary tissues and tumors upon the application of radiation. It expands the responsiveness of tumors towards radiation more than that of normal and healthy tissue [41]. Until now, several NPs have been investigated as potential radio-sensitizers to improve radiation therapy [32]. By exploiting the EPR effect, nanoparticles preferentially permeate into and accumulate in the cancerous tissues. This permeation of NPs into the tumor interstitial liquid (TIF) is the consequence of vascular endothelium sores in strong tumors and retention of NPs in TIF results due to reduced lymphatic drainage [42]. The kinds of NPs that are right now being explored for malignant therapeutics are metallic NPs, carbon nanotubes, liposomes, micelles, polymeric NPs, protein NPs, ceramic NPs, viral NPs, dendrimers, etc. [43]. Recently, an increase has been observed in the utilization of formulas to redesign radiotherapeutic impacts, especially using metal-based nanoparticles [44]. The stuffed metal-based NPs can specifically disperse or potentially ingest the high-energy radiations resulting in more restricted and merged harm. Aside from metal nanoparticles, various other nanoparticles and nanomaterials are being taken advantage of including quantum dots, metal oxides, super magnetic iron oxides, and numerous other non-metal-based nanoparticles [30].

#### 4.2.1 Metal-based nanoparticles

The conveyance of a deadly portion of radiation to the tumor cells and staying away from collateral cells from harm is the greatest challenge in radiotherapy. There has been a great interest in the advancement of radiosensitizing agents to work on the helpful proportion of radiotherapy. One exceptional system is to build the radiation portion inside tumors using metals with high atomic numbers (Z) [45]. Metal nanoparticles are utilized broadly in radiotherapy which builds the explicitness of radiation to designated sites diminishing the portion of radiation and staying away from toxicity and harm to the ordinary tissues [46].

The radiation-sensitizing impact of high atomic number (high-Z) NPs is based on the photoelectric ingestion of the material, which can actuate the arrival of photons or electrons [47]. Upon application of radiation, a bound electron consumes a photon and gets ejected from the particle. The cross-segment, or cooperation likelihood, of the photoelectric assimilation, relies upon the atomic number [48]. This implies that elements with a high Z have a higher likelihood to produce optional radiation. The radiated Auger electrons incite radiolysis of the encompassing water molecules and the production of ROS such as oxidizing free hydroxyl radicals, bringing about harm and cell death [49].

**Fig. 3:**
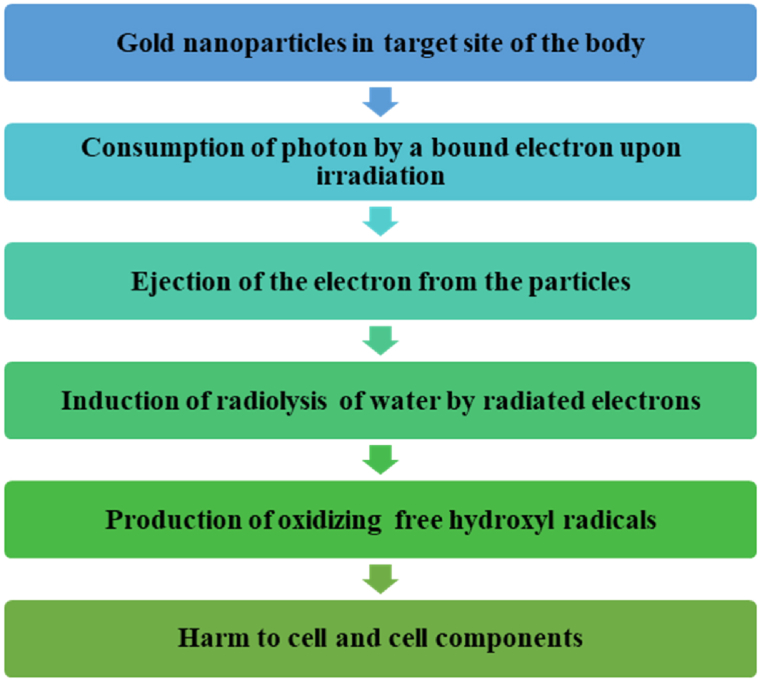
Mechanism of action of metal-based nanoparticles in case of nanotechnology-based radiation therapy.

**Fig. 4:**
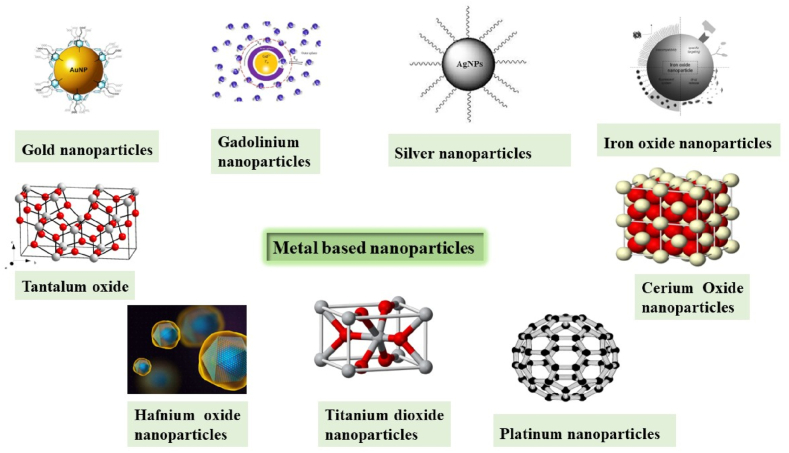
Different types of metal-based nanoparticles.

Metal nanoparticles, upon interaction with ionizing radiations, enhance the production of ROS within the tumors incrementing oxidative stress on the cancerous cells, promoting apoptotic cell death, and diminishing clonogenic endurance [50]. A flow chart for the mechanism of action of metal-based nanoparticles is shown in Fig. 3. Different metal nanoparticles, for example, gold, silver, platinum, zinc oxide, titanium dioxide, and so on are utilized for radiotherapy [51]. A list of metal-based nanoparticles and their beneficial properties to use for radiation therapy are shown in Table 1, Table 2 and their structural appearances are shown in Fig. 4.

**Table 1:** Metal-based nanoparticles and their beneficial properties.

Nanoparticles	Beneficial Properties
Gold nanoparticles	Inert, Biocompatible, low systemic clearance, variable size
Gadolinium nanoparticles	Reduced toxicity, fast disposal through kidneys, higher neutron capturing
Silver nanoparticles	Cost-effective, Induction of increased production of ROS
Platinum nanoparticles	Induction of potential DNA damage in the presence of hadron radiation

**Table 2:** Metal-based nanoparticles and their beneficial properties with respect to nanotechnology bases radiation therapy.

Nanoparticles	Beneficial properties and uses
Titanium dioxide nanoparticles	Kill cancer cells utilizing photocatalytic chemistry
Hafnium oxide nanoparticles	Biocompatible, Show huge radio-sensitization for EBRT, Damage cell parts
Cerium oxide nanoparticles	Promote apoptosis of tumor cells only
Iron oxide nanoparticles	Have unique magnetic and plasmonic properties, Increase ROS in tumor cells
Tantalum oxide nanoparticles	Nontoxic, bio-latent, restrain cancer development upon X-ray light

##### 4.2.1.1 Gold nanoparticles

Gold nanoparticles (AuNPs) are important in the field of radiation oncology as a result of their optimal attributes. AuNPs are produced by the reduction of hydrogen tetrachloroaurate (HAuCl_4_). Reduction of Au^+3^ ions to gold atoms that are unionized prompts a supersaturated gold arrangement followed by gradual precipitation of gold as nanometer-sized particles, prompting gold colloids [52]. AuNPs are the best material for photosensitization as they are inert and profoundly biocompatible. Moreover, they lead to an impact of radiations over a huge area of tumor consequently circumventing the need of delivering nanoparticles to every cell of a tumor. When compared with low molecular contrast agents (such as iodine), AuNPs appear to have low systemic clearance which allows enough absorption of photosensitizing material into the tumor tissue [30]. The gold nanoparticles can vary in size or shape according to the delivery requirements of tumor tissue [53].

The radiosensitization impact of gold nanoparticles is accomplished mainly by improved associated damage to DNA [54]. AuNPs have been observed to initiate rapid fragmentation of DNA upon irradiation with X-rays. The Radiosensitization impact of AuNPs is additionally associated with the size of the particle and surface adjustment [55]. The local distribution of AuNPs inside tumors can affect their ability to radio-sensitization. When embedded in the lipid layer to prepare Au nano-capsules, AuNPs can be then distributed homogeneously inside the cytosol of the cell following endosomal escape [56]. Such homogeneous delivery of AuNPs inside cancer cells leads to a 3-fold expansion in radiation-induced cell and tissue damage [32]. Gold nanoparticles have also been shown to have applications in cancer theranostics, a combination of diagnosis and therapeutic approaches for cancer. These nanoparticles are excellent theranostic agents for carrier and synergistic photothermal and photodynamic therapies [57].

##### 4.2.1.2 Gadolinium nanoparticles

Gadolinium (Gd) nanoparticles or Gadolinium-based nanoparticles (GBN) have high (Z = 64) material which works on the adequacy of radiotherapy. Their clinical relevance is additionally supported by their reduced toxic effects and the rapid disposal of Gd-chelates through the kidneys [58]. Gd nanoparticles can radiate far off X-beams, γ-beams, Auger electrons, and inward changes over electrons while being lighted by neutrons. Gadolinium-based nanomaterials can also be used for enhanced neutron capture therapy [59]. Moreover, neutron capture therapy based on Gd has been demonstrated to be better compared to boron-based neutron capture therapy [25].

##### 4.2.1.3 Silver nanoparticles

Silver nanoparticles (AgNPs) have radio-sensitizing properties like gold nanoparticles [60]. Like other high Z-number atoms, AgNPs use a similar mechanism of action for radiosensitization. AgNPs cost comparatively less than gold nanoparticles and thus are cost-effective but comparatively less biocompatible [61]. AgNPs have been proven to increase oxidative stress in cancerous cells, affect membrane fluidity, and lead to apoptosis. The mechanism of radiosensitization in the case of AgNPs includes the arrival of Ag^+^ cations which capture the electrons and induce oxidative damage in the cells. They result in increased ROS production and reduction in intracellular ATP. A proapoptotic and antiproliferative impact on gliomas was observed after treating gliomas with such NPs followed by radiotherapy [62]. Recently, scientists have developed green synthesized AgNPs featured with biochemicals coating which furnish them with increased biological actions than chemically synthesized AgNPs [63]. Green-synthesized silver-gold nanoparticles coated by dopamine when exposed to near-infrared radiation induce photothermal therapy via apoptotic and necrotic pathways [64].

##### 4.2.1.4 Platinum nanoparticles

Platinum has a high nuclear number and has been used in radio-chemotherapy. The association of hadron radiation with platinum NPs showed a 2-times potentiation of DNA damage, likely connected with an *in situ* higher energy deposition [65].

##### 4.2.1.5 Metal oxide-based nanoparticles

Metal oxide-based nanoparticles especially the oxides of metals with high Z are additionally being used in upgrading the adequacy of the radiation treatment of cancer. Ordinarily utilized nanoparticles depend on Titanium dioxide, Hafnium oxide, Cerium Oxide, and so on.

##### 4.2.1.6 Titanium dioxide nanoparticles

Titanium dioxide nanoparticles are also valuable for destroying tumors using specific photocatalytic chemistry [30]. These nanoparticles, inside cancerous cells, lead to the production of ROS upon photoexcitation by UV radiation. Since UV radiations have limited penetration depth, these nanomaterials are less viable for deep-rooted cells and tissues in the body. Titanium nanoparticles have also been conjugated with gadolinium and further advanced with other rare earth metals in order to make them more susceptible to X-ray-based excitation [66].

##### 4.2.1.7 Hafnium oxide nanoparticles

Hafnium is another high-Z element investigated for its sensitivity to radio therapy. Hafnium oxide (HfO_2_) NPs have good biocompatibility and biodistribution and have shown huge radio-sensitization for EBRT [32]. They result in thermal stress and lead to damage to the cell parts [67]. Hafnium oxide-based NPs are potentially being investigated in clinical trials for cancer treatment. A radiosensitizer called NBTXR3 is being investigated for its potential in improving the effects of radiotherapy for the treatment of neck and head cell carcinoma, cervical adenocarcinoma, esophageal adenocarcinoma, recurrent lung non-small cell carcinoma, pancreatic adenocarcinoma, and adult soft tissue sarcoma. Most of these studies are in phase I clinical trials but some of them are also in phase II clinical trials [68].

##### 4.2.1.8 Cerium Oxide nanoparticles

Cerium oxide nanoparticles (CNPs) are novel materials for radiosensitization and drug delivery for the treatment of cancer [69]. CNPs can be used to selectively instigate apoptotic cell death in cancer cells protecting surrounding healthy tissues from radiation and ultimate oxidative damage [70]. CNPs show proapoptotic, pro-oxidant, and anti-invasive effects in melanoma and squamous cell carcinoma of the skin by increasing oxidative stress levels leading to apoptosis of only cancer cells [71].

##### 4.2.1.9 Iron oxide nanoparticles

Iron oxide nanoparticles, because of their specific magnetic and plasmonic properties, have been broadly addressed in cancer treatment. In the presence of non-toxic radiations like Near Infrared (NIR), iron oxide nanoparticles induce oxidative stress that ultimately leads to the death of cancer cells [72]. Iron oxide nanoparticles with magnetic properties are used to reduce damage to healthy cells while changing radiant energy to heat or leading to ROS production in cancerous cells only [73].

##### 4.2.1.10 Tantalum oxide

Tantalum is a bio-inert element with no toxicity and is widely used in fake joints, stents, and other clinical implants. Ta has a large X-ray attenuation coefficient because of its high atomic number [74] and large X-ray attenuation ability. Tantalum oxide nanoparticles can act as a radio-sensitizer to deposit more radiation energy into tumors to enhance radiotherapy through Compton scattering and the Auger effect. Tantalum oxide NPs can restrain cancer development upon X-ray irradiation, compared with radiation treatment alone [32].

#### 4.2.2 Superparamagnetic iron oxide nanoparticles

**Fig. 5:**
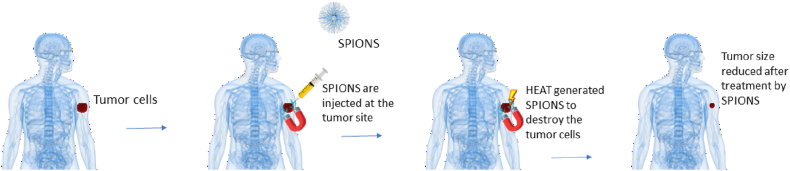
SPIONS to treat cancer cells.

Superparamagnetic iron oxide nanoparticles (SPOINs) predominantly include either maghemite (γFe_2_O_3_) or magnetite (Fe_3_O_4_). Because of their superparamagnetic properties, they can be directed, localized, and confined to a specific part of the body, by using external magnetic force [75], where they provoke localized hyperthermia or deliver specific agents resulting in radiation sensitization [29]. These nanoparticles lead to cytotoxic outcomes because of the development of ROS e.g., superoxide anion, hydroperoxyl radical, hydroxyl radical, and hydrogen peroxide which cause damage to DNA and other cell organelles [76] as depicted in Fig. 5. The iron superoxides have been utilized in a composition with other metal-based radiosensitizers like silver for therapeutic purposes [30].

#### 4.2.3 Metal alloy nanoparticles

Metal alloy nanoparticles are being exploited for their application in cancer radiation therapy and some of them have been shown to be effective radiosensitizers. Platinum-gold nanoparticles (Pt–Au NPs) functionalized with polyethylene glycol (PEG) have been developed that can selectively reside in a tumor via the EPR effect and enhances the efficiency of cancer radiotherapy with no significant side effects on normal tissues and organs [77]. Silver-gold nanoparticles (Au–Ag NPs) have also been developed and these nanoparticles showed an increased radiosensitization effect as compared to AuNPs and AgNPs. Au–Ag NPs showed increased radiation absorption and thus can enhance treatment effects with lower therapeutic radiation dosage [78].

#### 4.2.4 Quantum dots

Quantum dots are nanoparticles, which are made up of semiconductor materials (CaF, LaF, ZnS, or ZnO), that show quantum mechanical properties because of their size and are also capable of radio-sensitization [79]. The development and improvement of photosensitizing quantum dots is an extremely dynamic research area of interest. Photosensitizing quantum dots results in the generation of damage-causing radicals upon absorption of visible light. However, treatment modalities based on this mechanism are appropriate just for superficial cancer as visible light waves have little penetration depth [80]. After exposure to ionizing radiation like X-rays, these nanoparticles produce resolute luminescence or Auger electrons. Ionizing radiation has more penetration depth than visible light offering a critical benefit in the cancer therapy of patients with tumors in the internal organs [35].

#### 4.2.5 Carbon nanotubes

Recently the scientific community is also showing interest in carbon nanotubes. They have been studied for a variety of applications including drug delivery and biomedical imaging, however, their implication in radiation therapy is not very much investigated [81]. It is assumed that carbon nanotubes can reduce the radio-resistance of tumor cells by destroying DNA double-strands, promoting ROS generation, and down-regulating the cell cycle [82].

#### 4.2.6 Silica-based nanoparticles

Nanoparticles based on silica are also being tested for their potential role in radiosensitization. Though the simple silica-based NPs do not pose any substantial rise in oxidative damage, the amino silanized oxidized silicon nanoparticles result in higher ROS production upon irradiation and thus are more clinically useful than simple silica-based NPs [30].

#### 4.2.7 C_60_ nanoparticles

C_60_ is a fullerene with a unique globular structure containing 60 carbon atoms and consisting of 32 different member rings. Fullerene C_60_ induces certain markers of autophagy in cancerous cells and thus possesses potential anti-cancer activities [83]. Nanocrystals of underivatized fullerene C60, called Nano-C60, have been used in concentrations non-toxic to healthy cells and tissues in order to study their effects on radiosensitization. Cancerous cell lines treated with Nano-C60 *in vitro* upon irradiation with γ-radiation showed enhanced cell membrane damage and apoptotic cell death [84].

#### 4.2.8 Polymeric nanoparticles

Several NP formulations based on natural or synthetic polymers have been developed to enhance the effect of radiation or delivery of radiosensitizing agents [85]. Polyglycolic acid (PGA), Polylactic acid (PLA), and their copolymer Polylactic-co-glycolic acid (PLGA) based nanoparticles are being investigated for their application in radiation oncology [35]. *In vitro,* investigations have shown that treatment with paclitaxel PLGA results in the sensitization of tumor cell lines to radiation [86].

### 4.3 Challenges associated with the use of nanoparticles to enhance cancer radiation therapy

**Fig. 6:**
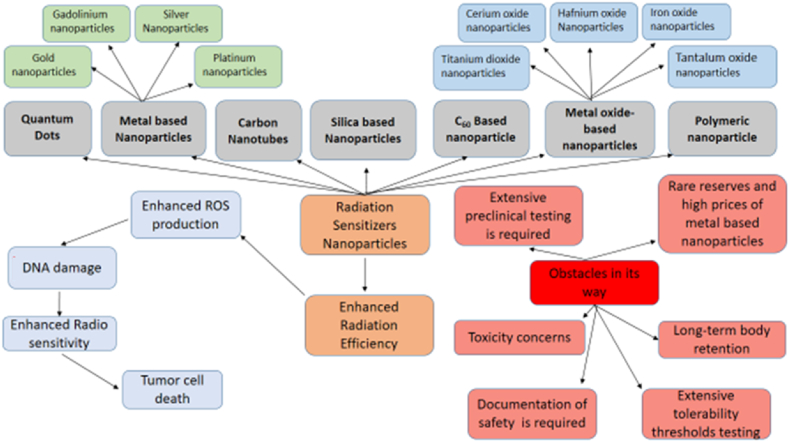
Overview of nanomaterial's used for radiation therapy and obstacles in its way.

During the last decades, several nanoparticles have been developed and explored for their ability to enhance the effect of radiation on tumors. However, there are certain challenges that are associated with the application of nanotechnology in the enhancement of cancer radiation therapy. One of the challenges regarding nanoparticles is the difficulty in obtaining appropriate physical, chemical, pharmacokinetic, and pharmacodynamic properties of the nanoparticles concerning size, mass, and specific shape. These properties are also associated with the secondary properties of nanoparticles such as toxicity and biocompatibility [87]. The effectiveness of the majority of nanoparticle formulations is influenced by certain biological challenges including modulating biodistribution as well as controlling nanoparticle permeation across several biological barriers [88]. Another main challenge regarding the clinical translation of nanotechnology-based radiation therapy is controlling the biological fate of the nanoparticles. One critical obstacle to using inorganic nanomaterials is their long-term retention in the body which raises serious concerns regarding their chronic toxicity as documented in Fig. 6. Scientists are focusing on engineering nanoparticles that are easily metabolizable, biodegradable, or renal clearable while maintaining their radiosensitization ability for enhanced radiation therapy to get rid of malignant tumors [89]. Furthermore, the focus is also on increasing the accumulation of the nanoparticles at the target site with high specificity decreasing off-target site accumulation.

Clinical translation of nanoparticles is also hindered by the difference in the information obtained regarding the efficacy and toxicity from preclinical studies in animals and clinical studies in humans [90]. This is particularly because of the heterogeneity of human disease and the general differences between animals and humans [91]. As expected, best-performing nanoparticle formulations in animal models during preclinical studies are likely to be investigated in clinical trials but the result is not always the same. Clinical translation of the nanoparticles also relies greatly on the consistency and reproducibility of the product. Nanoparticles that are used in preclinical studies are always synthesized in small batches. However, when it comes to clinical trials, challenges arise regarding their scale-up for large quantity synthesis, consistency, and reproducibility of the formulation [88].

**Fig. 7:**
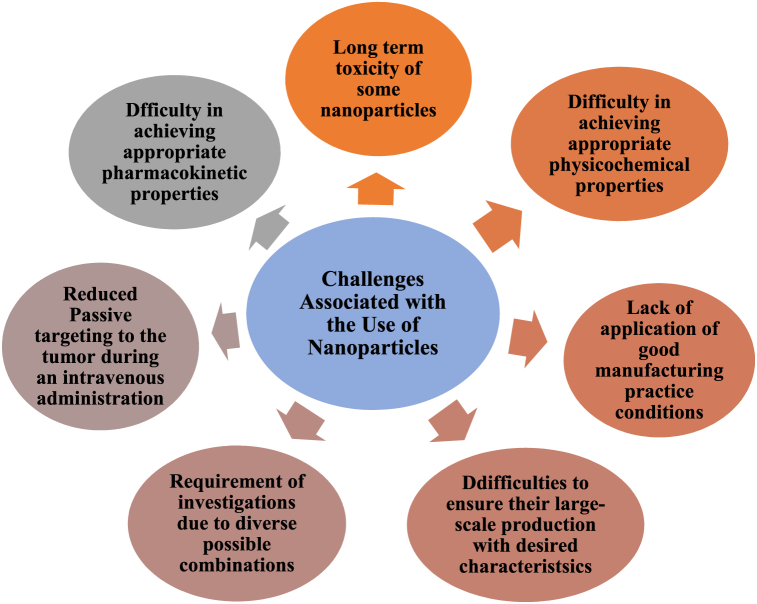
Challenges associated with the use of nanoparticles to enhance cancer radiation therapy.

Passive targeting of nanoparticles to the tumor during an intravenous administration is highly reduced by their rapid uptake by the spleen and liver and prolonged exposure to NP in these two organs coupled with their slow elimination can lead to chronic toxicity [92]. The toxicity of NP is a function of size, shape, concentration, surface functionalization, and surface charge [93]. The production of nanoparticles in a defined specification range is usually hindered by the fact that most procedures concerning nanoparticle preparations are not in coherence with good manufacturing practice (GMP) conditions [29]. Another challenge is the extensive range of systems that have been investigated crossing nanoparticle sizes, forms, shapes, surface charge, coating, concentration, etc. Because of the vast combination possibilities, it is difficult to investigate the mechanisms of uptake, the mechanism of radiation sensitization, the mechanisms of clearance from the body, and the mechanism of toxicity of nanoparticles with all possible combinations of properties [81]. A list of challenges associated with the use of nanoparticles for cancer radiation therapy is documented in Fig. 7.

A considerable number of radiosensitizers that are being used or investigated currently are based on elements with high prices and rare reserves, such as platinum and gold, which greatly limits their widespread use for clinical purposes [25]. Another challenge is to ensure immediate as well as long-term tolerability and safety of nanoparticles in humans. Certain metals, like gold, have been proven to be safe for clinical use in the treatment of a variety of diseases, other than cancer, like arthritis. However, each new nanoparticle-based on a different metal will require extensive preclinical testing. Moreover, it will also require proper documentation of tolerability and safety thresholds before advancing to early clinical trials. As part of this process, the biocompatibility, biodistribution, pharmacokinetics, and pharmacodynamics of the newly developed nanoparticles need to be thoroughly investigated [38].

Only a few nanoparticles are available in the market due to the difficulties in ensuring their large-scale production with adapted characterizations and the high production and characterization cost [27]. Cost vs benefit analysis thus needed to be checked before moving forward. Despite extensive preclinical studies, nanoparticle-based radiation therapy for cancer treatment has not yet been translated into the clinic. Despite sophisticated designs and formulations and excellent preclinical performance, most nanoparticles employed for radiotherapy to date demonstrate suboptimal pharmacokinetics *in vivo* leading to several toxicity concerns [37]. Moreover, the characterization of the nanoparticles used in radiotherapeutics is not always thorough. Hence, standardization of chemical and physical characterization of NPs needs to be carried out to further improve and facilitate the comparison between studies [94].

In a short period, the exploitation of radiation oncology has made a huge impact on cancer therapy. Today, several diagnostics and therapeutics based on nanotechnology are under clinical development. However, we are still in the initial stages of development of nanotechnology-based therapeutic techniques for cancer treatment, and by taking advantage of new technical capabilities offered by nanotechnology, we can further improve the therapeutic efficacy of radiation therapy, reduce toxicity, and enable more personalized treatment [36].

### 4.4 Future perspectives

Researchers are actively working on developing nanoparticles that can enhance the sensitivity of cancer cells to radiation. Future advancements may involve fine-tuning the properties of nanoparticles to maximize their radiation-sensitizing capabilities and improve overall treatment outcomes. Scientists are also working on the development of various nanoparticles that can deliver radiation and provide real-time imaging feedback on tumor response. This would allow clinicians to monitor treatment efficacy and make adjustments as needed, leading to more targeted and effective therapies. Moreover, for nanoparticle-based radiation therapy to become a standard clinical approach, regulatory approval, and clinical translation are crucial. Extensive preclinical and clinical studies are needed to establish safety, efficacy, and long-term effects. Additionally, optimizing manufacturing processes and scalability are required for the widespread adoption and integration of nanoparticle-based radiation therapy into clinical practice.

## 5 Conclusion

Nanotechnology is widely being used to enhance the efficacy and efficiency of radiation therapy for effective cancer treatment. Unique chemical, physical and biological properties of nanoparticles have allowed them to be utilized to increase the radiation response and overcome the tolerance of cancerous cells towards radiation and several other challenges associated with classical radiation therapy. Several nanoparticles have been investigated for their potential role as radiosensitizers and good results have been observed in this regard as the majority of nanoparticles have been shown to enhance radiation therapy by increasing oxidative stress, membrane fluidity, and apoptosis of cancerous cells. However, certain nanoparticles such as carbon nanotubes have been less investigated in this regard. Although nanotechnology has helped to overcome the problems associated with classical radiation therapy, the use of nanoparticles themselves has led to certain other challenges. High and prolonged exposure to nanoparticles in certain organs can lead to chronic toxicity. Moreover, it is also difficult to achieve appropriate physicochemical and pharmacokinetic in a defined specification range. In nanotechnology several combinations of different nanoparticles are possible and each of the combinations needs to be separately observed and investigated in the preclinical and clinical trials for the best results. In short, still, a lot of research is required in the field prior to its clinical manifestation. Scientists are focusing on overcoming the challenges associated with the use of nanoparticles as well e.g., scientists are focusing on engineering nanoparticles that are biodegradable, metabolizable, or renal clearable and they are trying to identify the mechanisms of uptake through physical and chemical characterization of nanoparticles. Despite several obstacles, nanotechnology has made a huge impact on radiation therapy in the field of oncology. However, we are still in the initial stages and there is a challenge to further improvements in the therapeutic efficacy of radiotherapy and to overcome its toxicity using the nanotechnology-based techniques.

## Author contribution statement

All authors listed have significantly contributed to the development and the writing of this article.

## Data availability statement

No data was used for the research described in the article.

## Declaration of competing interest

The authors declare that they have no known competing financial interests or personal relationships that could have appeared to influence the work reported in this paper.
